# Efficacy and safety of acupoint catgut embedding for knee osteoarthritis: A systematic review and meta-analysis

**DOI:** 10.1097/MD.0000000000046676

**Published:** 2025-12-19

**Authors:** HuiYan Zhao, Yan Liu, Changsop Yang, JaeWan Lee, Chang-Hyun Han

**Affiliations:** a KM Science Research Division, Korea Institute of Oriental Medicine, Daejeon, Republic of Korea; b Korean Convergence Medical Science, University of Science & Technology, School of Korea Institute of Oriental Medicine, Daejeon, Republic of Korea; c Department of Acupuncture, Beijing Guangyin Hospital, Chaoyang, Beijing, China; d St. Johnsbury Academy Jeju, Seogwipo-si, Republic of Korea.

**Keywords:** acupoint catgut embedding, knee osteoarthritis, meta-analysis, systematic review, thread embedding

## Abstract

**Background:**

Acupoint catgut embedding (ACE) is a therapeutic method for pain management that involves inserting a thread or catgut into the body with anti-hyperalgesic effect. This study aims to systematically review and comprehensively compare the effectiveness of ACE with that of each comparator in the treatment of knee osteoarthritis (OA).

**Methods:**

We searched 11 databases (PubMed, Embase, Cochrane Central Register of Controlled Trials, Web of Science, Chinese Biomedical Literature Database, Chinese Scientific Journal Database, WanFang Database, China National Knowledge Infrastructure, Oriental Medicine Advanced Searching Integrated System, Science-On, and KoreaMed) from their inception through August 1, 2023, without language limitations. Additionally, 2 registration platforms – ClinicalTrials.gov and the World Health Organization International Clinical Trials Registry – were searched for ongoing trials. The primary outcomes were assessed using a visual analog scale (VAS) and the Western Ontario and McMaster Universities OA Index. The secondary outcomes included the total effective rate, Lysholm score, and adverse effects. The risk of bias was assessed using the Cochrane handbook. The meta-analysis was performed using review manager, while the quality of evidence was evaluated using GradePro.

**Results:**

A total of 28 randomized controlled trials were included in the present study, encompassing 2120 participants. The most frequently used acupoint was GB34. The overall risk of bias was unclear. According to the meta-analysis results, the combination of ACE and conventional treatments showed higher effectiveness in term of the VAS, Western Ontario and McMaster Universities OA Index, total effectiveness, and Lysholm scores. Moreover, compared with the sham ACE control, the results showed that the combination of ACE and conventional treatments was more beneficial in term of the VAS, Western Ontario and McMaster Universities OA Index, and total effectiveness.

**Conclusion:**

Despite some potential improvement, the current evidence regarding the effectiveness of ACE for the treatment of knee OA is inconclusive due to the poor quality of the available evidence. Future well-designed randomized controlled trials are needed to confirm ACE’s effectiveness for treating knee OA.

## 1. Introduction

Knee osteoarthritis (OA), a common progressive multifactorial joint disease, can result in chronic pain and disability.^[1]^ There are 6 main risk factors for knee OA: obesity, various comorbidities (such as depression, chronic obstructive pulmonary disease, and hypertension), occupational factors (such as heavy lifting, frequent climbing, prolonged kneeling, squatting, standing, and physical activity), biomechanical factors (such as altered joint loading), and dietary exposure.^[2–4]^ Because there is a wide variety of risk factors, the number of patients with knee OA is rapidly increasing worldwide. From 1990 to 2019, the prevalence of knee OA was 4.90% (4711.88/1,00,000) for both males and females in 201 countries,^[5]^ while the proportion of the population over 45 years of age with knee OA is estimated to reach 29.5% from 2012 to 2032.^[6]^ Knee OA puts a high economic burden on the patient due to the ongoing treatment and care required,^[7]^ resulting in an urgent need to find suitable treatments for its management.

Knee OA is currently treated with conventional management (physical therapy, oral pain medicine), interventional management (intraarticular injection), surgery (knee replacement), cellular therapy (autologous chondrocyte implantation), and gene therapy.^[8]^ Considering that approximately 73% of individuals living with knee OA are over the age of 55 years, although physical therapy is needed, it would involve a high degree of self-reliance over the long period of progression.^[9]^ Regarding oral pain medicines, some medicines are associated with adverse reactions, such as the potential for indigestion associated with the use of nonsteroidal anti-inflammatory drugs.^[10]^ Approximately 2 to 5 years after knee replacement surgery, 15% of patients reported moderate or severe pain,^[11]^ while with cellular therapy, which is a newer approach to the treatment of knee OA, the incidences of mild and moderate adverse events reported were 89% and 11%, respectively.^[12]^ Therefore, the demand for complementary and alternative medicine (CAM) to be used in conjunction with conventional treatments in knee OA is increasing.

Acupoint catgut embedding (ACE), a type of CAM, provides long-term stimulation to an acupoint through an inserted thread or catgut.^[13]^ ACE has been widely used in many countries, including China, Korea, Mexico, and Indonesia.^[14–17]^ Due to the mechanism by which ACE functions to treat pain, ACE can exhibit antihyperalgesic effects by inhibiting the Sigma-1 receptor and decreasing the activation of the P38 mitogen-activated protein kinases in an inflammatory pain rat model.^[18]^ However, no systematic reviews have assessed ACE’s effects in treating knee OA. Therefore, in the present study, we performed a systematic review to evaluate all of the available evidence, determine the validity of using ACE in treating knee OA, and help practitioners make decisions about the best course of treatment for each patient. The 2 main research questions raised in the present study are as follows: Is ACE alone associated with a reduction in pain in patients with knee OA compared to conventional treatments? In clinical practice, is the combination of ACE with other conventional treatments a good choice for treating knee OA?

## 2. Materials and methods

### 2.1. Study registration

The present study followed the Preferred Reporting Items for Systematic Reviews and Meta-Analyses guidelines and has been registered in the International Prospective Register of Systematic Reviews (no. CRD42023454230).^[19]^ Additionally, the study protocol has been published previously.^[20]^

### 2.2. Data sources

The following 11 electronic databases were searched from inception through August 1, 2023, without language limitations: PubMed, EMBASE, Cochrane Central Register of Controlled Trials, Web of Science, Chinese Biomedical Literature Database, Chinese Scientific Journal Database (VIP Database), WanFang Database, China National Knowledge Infrastructure, Oriental Medicine Advanced Searching Integrated System, Science-On, and KoreaMed. To create a list of potentially missing eligible studies, we also searched ongoing trial databases from the World Health Organization International Clinical Trials Registry Platform (http://apps.who.int/trialsearch/Default) and ClinicalTrials.gov (http://ClinicalTrials.gov/). The following search terms were used: Knee OA (e.g., “osteoarthritis, knee” or “knee”); catgut embedding (e.g., “catgut” or “thread”); and randomized controlled trial (e.g., “random” or “randomized controlled trial”). All the search strategies utilized in the present study are listed in Table S2, Supplemental Digital Content, https://links.lww.com/MD/Q928 of our previous study.^[20]^

### 2.3. Inclusion criteria for study selection

We included all randomized controlled trials (RCTs) on ACE for the treatment of patients with knee OA of any age, race, or sex, who were diagnosed with knee OA using tools, such as the American College of Rheumatology/Arthritis Foundation guidelines for the diagnosis and treatment of OA^[21]^ or other valid diagnostic criteria. The intervention types were ACE alone or in combination with conventional treatments, with no restrictions on the type of ACE, and comparisons included possible original treatments and placebos or controls. The visual analog scale (VAS)^[22]^ and Western Ontario and McMaster Universities OA Index (WOMAC)^[23]^ were used as the primary outcomes, while the secondary outcomes were the total effective rate (based on the CAM score questionnaire^[24]^), Lysholm score,^[25]^ and adverse events.

### 2.4. Data extraction and quality assessment

Two authors (HYZ and YL) independently screened and read the articles, and any disagreements were resolved by consensus with a third reviewer (CHH). The 2 authors (HYZ and YL) then independently extracted the data using a standard data extraction form, which included the following: the first author, year of publication, country, method of intervention and control (acupoint treated and type of ACE), treatment duration, outcome measurements, and adverse events. The extracted data were then entered into an Excel spreadsheet (Microsoft Office Professional Plus 2019; Microsoft, Albuquerque). Any concerns were, again, addressed by consensus with a third reviewer (CHH). For missing data, questionable data, and ongoing trials, we requested clarification from the corresponding authors by e-mail. If the corresponding author failed to respond, the study was then excluded.

The Cochrane Handbook for Systemic Reviews of Interventions classifies the risk of bias based on 7 criteria^[26]^: selection bias (random sequence generation and allocation concealment), performance bias (blinding of participants and personnel), detection bias (blinding of outcome assessment), attrition bias (incomplete outcome data), reporting bias (selective outcome reporting), and other biases.^[27]^ Following the aforementioned criteria and other biases, we assessed the method of sample size calculation. The entire process was performed independently by 2 reviewers (HYZ and YL), and any disagreements were resolved by consensus with a third reviewer (CHH).

The Grading of Recommendations Assessment, Development, and Evaluation method was used to assess the quality of evidence for all outcomes.^[28]^ This method includes 5 major domains (consistency, limitations, imprecision, indirectness, and publication bias) and 4 levels of evidence quality (high, moderate, low, and very low).

### 2.5. Data synthesis

Continuous data were analyzed using mean difference (MD). For dichotomous data, we summarized risk ratios (RRs) using 95% confidence intervals (CIs). Review Manager version 5.4 from Cochrane Collaboration (RevMan 2020; Cochrane Collaboration, London, England) was used for the data analysis. Statistical heterogeneity was analyzed using *I*^2^ and *χ*^2^ tests, and the status of heterogeneity was categorized based on the percentage obtained (0–50%, low; 50–75%, serious; >75%, very serious). Due to the type of ACE, most studies used a random-effects model; however, if *P* > .1 and *I*^2^ < 50%, we used the fixed-effects model. The meta-analysis compared ACE versus conventional treatments, ACE + conventional treatments versus conventional treatments, and ACE + conventional treatments versus sham ACE + conventional treatments. We subsequently performed a subgroup analysis based on the characteristics of the study design.

## 3. Results

**Figure 1.**
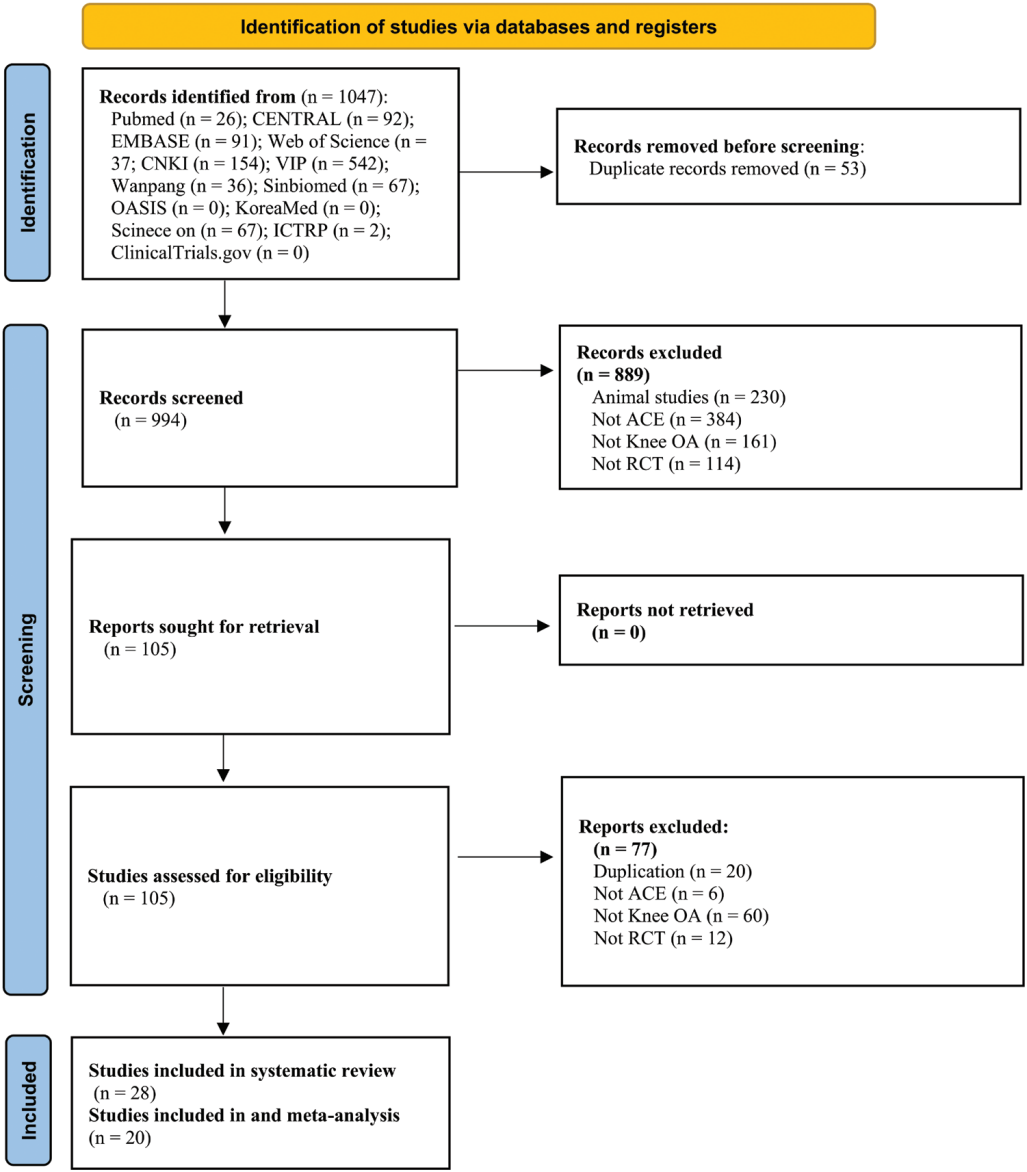
Flow chart of the study selection process. ACE = acupoint catgut embedding, CENTRAL = Cochrane Central Register of Controlled Trials, CNKI = China National Knowledge Infrastructure, ICTRP = International clinical trials registry platform, OA = osteoarthritis, OASIS = oriental medicine advanced searching integrated system, RCT = randomized controlled trials.

A total of 1047 articles were extracted during the initial screening of the electronic databases: PubMed, 26; EMBASE, 91; Cochrane Central Register of Controlled Trials, 92; Web of Science, 37; China National Knowledge Infrastructure, 154; VIP, 542; WanFang, 36; Chinese Biomedical Literature Database, 67; Oriental Medicine Advanced Searching Integrated System, 0; Science-On, 67; KoreaMed, 0; Clinicaltrials.gov, 0; and International Clinical Trials Registry Platform, 2. After removing duplicate studies, 994 remained, and after the first screening based on title and abstract, 105 remained, which were selected for full-text review. After the full-text review, 77 studies were excluded because they did not meet the eligibility criteria. Therefore, a total of 28 RCTs (including one three-armed study) were included in the present systematic review; however, as 2 studies belonged to ongoing trials and 6 studies did not assess the primary and secondary outcomes, only 20 RCTs were included in the present meta-analysis. The process of screening is shown in Figure 1.

### 3.1. Study characteristics

The characteristics of the 28 included studies are listed in Table 1. In brief, 3 studies were conducted in Korea, 1 in Iran, and 24 in China, while 4 were written in English and 24 in Chinese. A total of 2120 patients with knee OA were included in the present systematic review, with sample sizes ranging from 12 to 200 and treatment durations ranging from 3 weeks to 6 months. Of the 28 RCTs, 24 reported acupoints, the frequencies of which were as follows: GB34, 17 times; ST34, 16 times; SP10, 14 times; SP9, 10 times; ashi, 7 times; EX-LE and ST36, 6 times; BL11 and EX-LE5, 4 times; BL40 and ST35, 3 times; BL17, EX-LE4, GB30, and RN17, 2 times; and BL21, BL24, BL39, BL57, EX-B05, GB31, KI10, LR3, LR8, LR9, SP09, and SP11, 1 time.

**Table 1:** Characteristics of included studies.

No	Author (yr) country	Experimental intervention (sample size)	Acupoints of ACE	Type of ACE	Control intervention (sample size)	Treatment duration	Outcome measure	Effect estimates	Adverse events
1	Bahrami (2023) Iran	(A) ACE (24)	n.r.	Catgut	(B) TENS (50–100 Hz) (24) (C) WM (Iuprofen, 400 mg, twice a d) (24)	8 wk	1) VAS 2) IL-6 3) WOMAC 4) Quality of Life	Not available	Not available
2	Cheng (2018) China	(A) ACE (50)	KunGua, GenGua, KanGua, Zhengua, SP 10, ST34, SP 9, GB34, Ex-B05	n.r.	(B) Acupoint-injection (Sodium Hyaluronate) plus WM (Meloxicam, 7.5 mg, 2 times a day), HM (DuHuo JiSheng Tang), and usual care (50)	6 mo	1) HSS 2) BBS 3) Total effective rate (TM score 1)	1) MD, 3.40 [0.81, 5.99], *P* = .01 2) MD, 6.10 [2.98, 9.22], *P* = .0001 3) RR, 1.15 [0.99, 1.33], *P* = .07	n.r.
3	Chen (2018) China	(A) ACE (30)	Ashi acupuncture point	PDO	(B) MA (30)	4 wk	1) VAS 2) WOMAC	1) MD, −0.60 [−1.25, 0.05], *P* = .07 2) Joint stiffness: MD, −1.13 [−1.88, −0.38], *P* = .003; physical function: MD, −7.43 [−11.60, −3.26], *P* = .0005; pain: −0.54 [−1.65, 0.57], *P* = .34	Dizziness (1)
4	Dong (2018) China	(A) ACE plus Practice (30)	BL11, BL21, GB34, LR3	n.r.	(B) Sham embedding plus practice (30)	4 wk	1) NRS 2) WOMAC 3) TM score 1 4) IL-1 5) MMP-13 6) loxoprofen sodium tablets usage 7) Total effective rate (NRS) 8) Total effective rate (WOMAC) 9) Total effective rate (TM score 1)	1) MD, −2.10 [−2.76, −1.44], *P* < .00001 2) MD, −30.93 [−38.10, −23.76], *P* < .00001 3) MD, −3.40 [−4.48, −2.32], *P* < .00001 4) MD, −16.54 [−42.87, 9.79], *P* = .22 5) MD, −43.01 [−138.63, 52.61], *P* = .38 6) MD, −0.50 [−0.77, −0.23], *P* = .00003 7) RR, 5.40 [2.40, 12.13], *P* < .0001 8) RR, 3.59 [2.02, 6.37], *P* < .0001 9) RR, 1.42 [1.12, 1.80], *P* = .004	n.r.
5	Guo (2016) China	(A) ACE plus (B) (58)	n.r.	Catgut	(B) WM (Glucosamine Hydrochloride Capsules) (58)	4 wk	1) SF-36 2) Total effective rate (Lysholm score, VAS)	1) Physical functional: MD, 14.34 [9.88, 18.80], *P* < .00001; role physical: MD, 9.57 [2.88, 16.26], *P* = .005; bodily pain: MD, 23.43 [18.05, 28.81], *P* < .00001; general health: MD, 7.64 [2.68, 12.60], *P* = .003 2) RR, 1.11 [0.94, 1.32], *P* = .23	n.r.
6	Hou (2010) China	(A) ACE (n.r.) plus arthroscopic debridement of articular cavity, practice, and HM (HuoYu, XiaoYu, JuoBi Tang) (26)	ST36	n.r.	(B) Arthroscopic debridement of articular cavity plus practice and WM (Fenbid; 1 pill; 2 times a wk) (26)	2 wk	1) Lysholm score 2) Total effective rate (Lysholm score)	1) MD, 19.90 [15.04, 24.76], *P* < .00001 2) RR, 1.25 [1.00, 1.56], *P* = .05	n.r.
7	He (2021) China	(A) ACE plus (B) (31)	n.r.	Catgut	(B) WM (glucosamine hydrochloride capsules) (31)	1 mo	1) VAS 2) Biofunction 3) Health 4) Total effective rate	1) MD, −0.88 [−0.96, −0.80], *P* < .00001 2) MD, 9.11 [7.57, 10.65], *P* < .00001 3) MD, 10.43 [8.70, 12.16], *P* < .00001 4) RR, 1.36 [1.08, 1.72], *P* = .009	n.r.
8	Huang (2021) China	(A) ACE plus WA (60)	QuanXi Xue (Shen Xue, Tui Xue)	Catgut	(B) WM (Diclofenac Sodium extended release tablets) (58)	4 wk	1) VAS 2) Lysholm score 3) WOMAC 4) TM score 1 5) IL-1 6) IL-6 7) TNF-α	1) MD, −1.45 [−1.57, −1.33], *P* < .00001 2) MD, 14.12 [11.19, 17.05], *P* < .00001 3) Pain: MD, −1.59 [−1.75, −1.43], *P* < .00001; Joint stiffness: MD, −0.71 [−0.78, −0.64], *P* < .00001; physical function: MD, −4.98 [−5.83, −4.13], *P* < .00001 4) Pain while lying in bed at night: MD, −0.64 [−0.69, −0.59], *P* < .00001; morning stiffness: MD, 0.69 [0.62, 0.76], *P* < .00001; walking: MD, −0.74 [−0.79, −0.69], *P* < .00001; pain from standing up from sitting position: MD, −0.71 [−0.76, −0.66], *P* < .00001; maximum walking distance: MD, −0.27 [−0.30, −0.24], *P* < .00001; daily exercise: MD, −0.34 [−0.37, −0.31], *P* < .00001; go upstairs: MD, −0.31 [−0.34, −0.28], *P* < .00001; down stairs: MD, −0.23 [−0.26, −0.20], *P* < .00001; bend the knee: MD, −0.20 [−0.22, −0.18], *P* < .00001 5) MD, −3.92 [−4.45, −3.39], *P* < .00001 6) MD, −21.20 [−23.12, −19.28], *P* < .00001 7) MD, −3.31 [−4.10, −2.52], *P* < .00001	n.r.
9	Huang (2022) China	(A) ACE (25)	GB34, SP9, ST36, SP10, ST34, EX-LE2	Catgut	(B) Acupoint-injection (Sodium Hyaluronate) (26)	1 mo	1) WOMAC 2) IL-1 3) TNF-α 4) Total effective rate (TM score 1)	1) MD, −4.45 [−8.92, 0.02], *P* = .05 2) MD, −2.46 [−17.11, 12.19], *P* = .74 3) MD, −0.06 [−0.18, 0.06], *P* = .31 4) RR, 1.09 [0.86, 1.38], *P* = .48	n.r.
10	Jang (2023) Korea	(A) ACE (30)	ST34, SP10, EX-LE4, ST35, SP9, GB34, BL40, LR9, BL24, and GB30	PDO	(B) MA (30)	4 wk	1) WOMAC 2) VAS 3) EQ-5D 4) ROM 5) Patient global impression of change 6) Dosage of rescue medication	Not available	Not available
11	Lee (2022) Korea	(A) ACE plus usual care and EA (6)	EX-LE4, ST35, EX-LE5, EX-LE2, ST34, SP10, ST36, GB34, SP9, LR8, BL40	PDO	(B) Usual care (6)	4 wk	1) VAS 2) WOMAC 3) EQ-5D 4) Medication consumption	1) MD, −24.50 [−42.90, −6.10], *P* = .009 2) Total: MD, −15.66 [−39.81, 8.49], *P* = .20; pain: MD, −3.50 [−8.54, 1.54], *P* = .17; joint stiffness: MD, −1.00 [−2.99, 0.99], *P* = .33; physical function: −11.17 [−29.22, 6.88], *P* = .23 3) MD, 0.08 [−0.12, 0.28], *P* = .43 4) MD, −6.84 [−20.30, 6.62], *P* = .32	None
12	Lei (2022) China	(A) ACE plus WM (nonsteroidal anti-inflammatory drug) (43)	Ashi acupuncture point	PDO	(B) Sham embedding plus WM (nonsteroidal anti-inflammatory drug) (43)	4 wk	1) HSS 2) VAS 3) WOMAC 4) Total effective rate (TM score 2)	1) MD, 6.19 [3.98, 8.40], *P* < .00001 2) MD, −1.95 [−2.03, −1.87], *P* < .00001 3) Pain: MD, −3.89 [−4.79, −2.99], *P* < .00001; joint stiffness: MD, −1.04 [−1.17, −0.91], *P* < .00001; physical function: MD, −11.23 [−12.74, −9.72], *P* < .00001 4) RR, 1.17 [1.00, 1.37], *P* = .05	Rash (1), gastrointestinal dysfunction (1)
13	Lin (2020) China	(A) ACE plus (B) (30)	ST36, ST 34, SP10, SP9, GB34	PGLA	(B) Heated needle acupuncture (30)	5 wk	1) WOMAC 2) TM score 1 3) Total effective rate (TM score 1)	1) Pain: MD, −1.00 [−1.93, −0.07], *P* = .04; joint stiffness: MD, −0.40 [−0.73, −0.07], *P* = .02; physical function: MD, −3.63 [−6.64, −0.62], *P* = .02 2) Pain while lying in bed at night: MD, −0.27 [−0.47, −0.07], *P* = .008; morning stiffness: MD, −0.47 [−0.78, −0.16], *P* = .003; walking: MD, −0.44 [−0.83, −0.05], *P* = .03; pain from standing up from sitting position: MD, −0.30 [−0.80, 0.20], *P* = .24; maximum walking distance: MD, −0.20 [−0.42, 0.02], *P* = .07; daily exercise: MD, −0.30 [−0.90, 0.30], *P* = .32; go upstairs: MD, −0.30 [−0.58, −0.02], *P* = .04; down stairs: MD, −0.30 [−0.58, −0.02], *P* = .04; bend the knee: MD, −0.57 [−0.93, −0.21], *P* = .002; walking on uneven roads: MD, −0.43 [−0.82, −0.04], *P* = .03; total: MD, −3.57 [−6.42, −0.72], *P* = .01 3) RR, 1.04 [0.92, 1.16], *P* = .55	n.r.
14	Mai (2014) China	(A) ACE plus (B) (60)	GB34, SP9, ST36, SP10, ST34, EX-LE2, ST35	PGLA	(B) Acupoint-injection (Sodium Hyaluronate, 20 mg, once a wk) (60)	5 wk	1) HSS 2) Total effective rate (HSS)	1) Pain: MD, 1.65 [−1.73, 5.03], *P* = .34; function: MD, 1.32 [−1.21, 3.85], *P* = .31; range: MD, 1.91 [−0.71, 4.53], *P* = .15; power of muscle: MD, 1.38 [−0.79, 3.55], *P* = .21; flexion deformity of knee: MD, 1.45 [−0.85, 3.75], *P* = .22; stable, MD, 1.46 [−0.50, 3.42], *P* = .14 2) RR, 1.15 [0.98, 1.34], *P* = .08	n.r.
15	Mo (2017) China	(A) ACE plus acupoint application (36)	GB34, SP 09, ST 36, SP 10, ST 34, EX-LE2	Catgut	(B) Acupoint-injection (Lidocaine 5 mL, Triamcinolone Acetonide Injection 40 mg) (35)	8 wk	1) JOA 2) Total effective rate (JOA)	1) MD, 2.33 [−2.33, 6.99], *P* = .33 2) RR, 1.00 [0.87, 1.15], *P* = .97	None
16	Pan (2020) China	(A) ACE (35)	SP10, ST 34, SP9, GB34, EX-LE5, Ashi acupuncture point	PGLA	(B) MA (35)	3 wk	1) VAS 2) KOOS	1) MD, −0.25 [−0.59, 0.08], *P* = .13 2) MD, 1.17 [−1.70, 4.04], *P* = .42	n.r.
17	Shu (2022) China	(A) ACE (43)	Ashi acupuncture point	PDO	(B) MA (43)	4 wk	1) HSS 2) VAS 3) WOMAC 4) Total effective rate (TM score)	1) MD, 6.19 [3.98, 8.40], *P* < .00001 2) MD, −1.95 [−2.03, −1.87], *P* < .00001 3) Pain: MD, −3.89 [−4.79, −2.99], *P* < .00001; joint stiffness: MD, −1.04 [−1.17, −0.91], *P* < .00001; physical function: MD, −11.23 [−12.74, −9.72], *P* < .00001 4) RR, 1.17 [1.00, 1.37], *P* = .05	Rash (1), gastrointestinal dysfunction (1)
18	Tian (2022) China	(A) ACE plus (B) (62)	BL11	PGLA	(B) WM (Celecoxib, 0.1 g/one time, 2 times a day plus glucosamine, 0.75 g/one time, 2 times a d) plus hyperbaric oxygen (1 times a d) (62)	4 wk	1) WOMAC 2) NRS 3) Lequesne score (12 mo) 4) Total effective rate (TM score 1)	1) MD, −3.78 [−6.47, −1.09], *P* = .006 2) MD, −0.17 [−0.27, −0.07], *P* = .001 3) MD, −0.57 [−0.96, −0.18], *P* = .004 4) RR, 1.11 [0.99, 1.25], *P* = .07	n.r.
19	Wang (2021) 1 China	(A) ACE plus practice (55)	EX-LE2, ST34, SP9, GB34, BL40, ashi acupuncture point	Catgut	(B) ACE (55)	21 d	1) Total effective rate (TM score 2)	1) RR, 1.19 [1.01, 1.41], *P* = .04	n.r.
20	Wang (2021) 2 China	(A) ACE plus acupotomy therapy (30)	SP10, ST34, GB34, Ashi acupuncture point	PGLA	(B) MA (30)	4 wk	1) VAS 2) Total effective rate (TM score 1) 3) Lequesne score	1) MD, −0.93 [−1.04, −0.82], *P* < .00001 2) RR, 1.17 [0.95, 1.43], *P* = .14 3) MD, −2.70 [−5.03, −0.37], *P* = .02	None
21	Wang (2021) 3 China	(A) ACE plus acupotomy therapy (31)	SP10, ST34, EX-LE5, GB34	PGLA	(B) MA (31)	3 wk	1) VAS 2) Total effective rate (TM score 2)	1) MD, −3.93 [−5.32, −2.54], *P* < .00001 2) RR, 1.17 [0.93, 1.46], *P* = .17	n.r.
22	Wang 4 (2021) China	(A) ACE plus acupotomy therapy (35)	SP10, ST34, GB34	PGLA	(B) MA (35)	3 wk	1) PPI 2) VAS 3) SF-MPQ 4) PRI 5) Total effective rate (TM score 2)	1) MD, −0.32 [−0.68, 0.04], *P* = .08 2) MD, −0.74 [−1.48, −0.00], *P* = .05 3) MD, −2.85 [−5.12, −0.58], *P* = .01 4) Emotion: MD, −0.89 [−1.42, −0.36], *P* = .009; feeling: MD, −1.04 [−1.90, −0.18], *P* = .02 5) RR, 1.14 [0.94, 1.39], *P* = .18	n.r
23	Woo (2022) Korea	(A) ACE (18)	ST34, SP10, Medial collateral ligament, SP9, Side of patella, Lateral collateral ligament, Lateral joint line, Medial joint line	PDO	(B) MA (19)	6 wk	1) WOMAC 2) VAS 3) PPI 4) SF-MPQ	1) MD, 1.85 [−7.24, 10.94], *P* = .69 2) MD, 0.62 [−0.26, 1.50], *P* = .17 3) MD, −0.15 [−0.59, 0.29], *P* = .51 4) MD, −0.10 [−3.14, 2.94], *P* = .95	Mild pain, edema, bruising, and itching, sore throat, toothache, pain in the abdomen, lower back, shoulder, neck, and fingers
24	Yang (2016) China	(A) ACE (30)	BL11, BL17, RN17, GB34, SP11, ST34, EX-LE5, Ashi acupuncture point	PGLA	(B) MA (30)	1 mo	1) VAS 2) Total effective rate (TM score 1)	1) MD, −3.14 [−3.84, −2.44], *P* < .00001 2) RR, 1.08 [0.88, 1.32], *P* = .45	None
25	Yang (2017) China	(A) ACE (30)	BL11, BL17, RN17, GB34	n.r.	(B) MA (30)	2 mo	1) SF-36 2) Total effective rate (Re-found)	1) Physical functional: MD, 4.28 [2.65, 5.91], *P* < .00001; bodily pain: MD, −2.71 [−3.39, −2.03], *P* < .00001; general health: MD, −4.08 [−5.26, −2.90], *P* < .00001 2) RR, 0.21 [0.07, 0.67], *P* = .008	n.r.
26	Yang (2023) China	(A) ACE plus WA (51)	QuanXi Xue (Shen Xue, Tui Xue)	Catgut	(B) WM (diclofenac sodium sustained-release tablet) (51)	21 d	1) WOMAC 2) VAS 3) TM score 1 4) Total effective rate*	1) Pain: MD, −1.81 [−2.02, −1.60], *P* < .00001; joint stiffness: MD, −0.93 [−1.06, −0.80], *P* < .00001; physical function: MD, −5.18 [−6.13, −4.23], *P* < .00001 2) MD, −1.67 [−1.84, −1.50], *P* < .00001 3) Pain while lying in bed at night: MD, −0.84 [−0.93, −0.75], *P* < .00001; morning stiffness: MD, −0.91 [−1.00, −0.82], *P* < .00001; walking: MD, −0.96 [−1.05, −0.87], *P* < .00001; pain from standing up from sitting position: MD, −0.93 [−1.03, −0.83], *P* < .00001; maximum walking distance: MD, −0.49 [−0.56, −0.42], *P* < .00001; daily exercise: MD, −0.56 [−0.64, −0.48], *P* < .00001; go upstairs: MD, −0.53 [−0.60, −0.46], *P* < .00001; down stairs: MD, −0.14 [−0.21, −0.07], *P* < .0001; bend the knee: MD, −0.20 [−0.27, −0.13], *P* < .00001 4) RR, 1.26 [1.06, 1.50], *P* = .009	n.r.
27	Yu (2022) China	(A) ACE (100)	GB 31, ST 34, SP 10, EX-LE2, BL39, KI10, SP9, GB34, BL57, GB30	PGLA	(B) Acupoint-injection (Sodium Hyaluronate) plus WM (Diacerein Capsules, 1 capsule/1 time, 2 times a d) (100)	12 wk	1) WOMAC 2) ROM 3) Total effective rate (TM score 1)	1) Pain: MD, 0.13 [−0.32, 0.58], *P* = .57; physical function: MD, 1.65 [−1.12, 4.42], *P* = .24; joint stiffness: MD, 0.13 [−0.32, 0.58], *P* = .57; total: MD, 2.70 [−0.44, 5.84], *P* = .09 2) MD, −0.36 [−1.71, 0.99], *P* = .60 3) RR, 0.94 [0.81, 1.09], *P* = .39	n.r.

* = no reference, ACE = acupoint catgut embedding, BBS = berg balance scale, EA = electro-acupuncture, EQ-5D = EuroQol-5Dimention, HM = herbal medicine, HSS = hospital for special surgery, IL-1 = interleukin-1, IL-6 = Interleukin-6, JOA: Japanese Orthopaedical Association, KOOS = knee injury and osteoarthritis outcome score, MA = manual acupuncture, MD = mean difference, MMP-13 = matrix metallopeptidase-13, n.r = not reported, NRS = numeric rating scale, PDO = polydioxanone, PGLA = polyglactin acid, PPI = present pain intensity, PRI = pain rating index, ROM = range of motion, RR = risk ratio, SF-36 = short form-36, SF-MPQ = short-form McGill pain questionnaire, TENS = transcutaneous electrical nerve stimulation, TM score 1 = guiding principles for clinical research of new traditional Chinese medicines, TM score 2 = standards for diagnostic and efficacy of traditional Chinese medicine diseases and syndrome, TNF-α = tumor necrosis factor-α, VAS = visual analog scale, WA = warm acupuncture, WOMAC = Western Ontario and McMaster Universities OA Index, WM = Western medicine.

The 28 included studies were divided into 17 groups based on the intervention and comparison groups: ACE versus manual acupuncture (MA; n = 7)^[17,29–34]^; ACE + warm-acupuncture versus Western medicine (WM; n = 3)^[35–37]^; ACE + WM versus WM (n = 2)^[38,39]^; ACE + acupotomy therapy versus MA (n = 3)^[40–42]^; ACE versus sham embedding (n = 2)^[43,44]^; ACE + usual care and electro-acupuncture versus usual care (n = 1)^[45]^; ACE + practice versus ACE (n = 1)^[46]^; ACE + acupoint application versus acupoint injection (n = 1)^[47]^; ACE versus acupoint injection (n = 1)^[48]^; ACE versus acupoint injection + WM, HM and usual care (n = 1)^[49]^; ACE versus acupoint injection + WM (n = 1)^[50]^; ACE versus WM (n = 1)^[51]^; ACE versus transcutaneous electrical nerve stimulation (n = 1)^[51]^; ACE + arthroscopic debridement of the articular cavity, practice, and HM versus arthroscopic debridement of the articular cavity + WM (n = 1)^[52]^; ACE + acupoint injection versus acupoint injection (n = 1)^[53]^; ACE + heated needle acupuncture versus heated needle acupuncture (n = 1)^[54]^; and ACE + WM and hyperbaric oxygen versus WM + hyperbaric oxygen (n = 1).^[55]^

### 3.2. Quality of included studies

**Figure 2.**
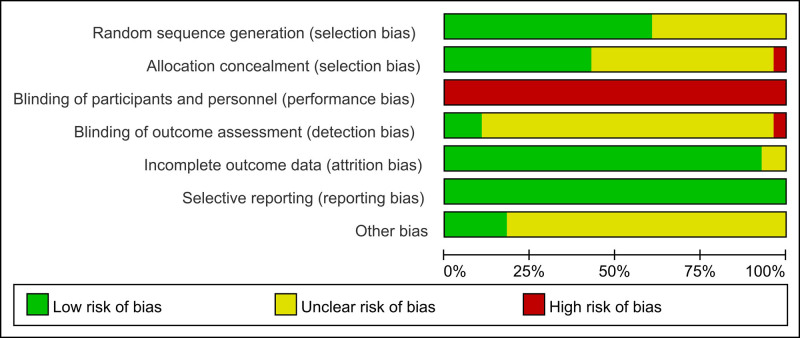
Risk of bias for included studies.

Figure 2 illustrates the risk of bias in the included studies. In brief, 17 studies mentioned randomization, which could be considered a low risk of bias in the random sequence generation part. For allocation concealment, 1 study^[53]^ used a visiting sequence that resulted in a high risk of bias, and 12 studies reported using computer programs to list random numbers, with a low risk of bias. Because it was impossible to blind the participants and practitioners in the RCTs, all trials were considered to have a high risk of bias. In regards to the outcome assessments (i.e., the questionnaire used), only 3 studies^[17,45,56]^ mentioned using a third researcher for performing the outcome assessments, which was considered a low risk of bias. All of the studies were considered to have a low risk of bias due to incomplete outcome data and selective reporting. Additionally, some studies^[17,40,45,56,57]^ reported a sample size calculation method and were considered to have a low risk of bias. The quality of each study is shown in Figure S1, Supplemental Digital Content, https://links.lww.com/MD/Q928.

### 3.3. Synthesis of results

#### 3.3.1. VAS

**Figure 3.**
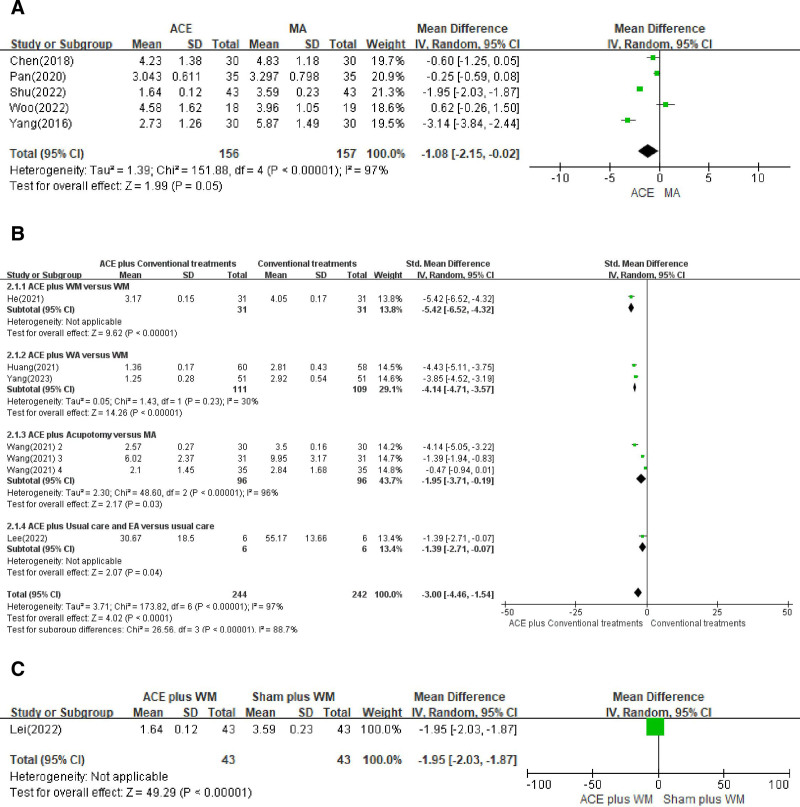
Forest plot of the visual analog scale scores in the comparison of ACE versus conventional treatments. (A) ACE versus MA, (B) ACE + conventional treatments versus conventional treatments, and (C) ACE + WM versus sham + WM. ACE = acupoint catgut embedding, CI = confidence interval, CT = conventional treatment, EA = electroacupuncture, MA = manual acupuncture, WA = warm acupuncture, WM = Western medicine.

ACE versus MA was included in the ACE versus conventional treatments analysis, and 5 studies^[17,29,31–33]^ out of 7, including 313 patients (156 in the ACE group and 157 in the MA group), were included in the meta-analysis to synthesize the VAS. As shown in Figure 3A, the pooled results indicated that ACE was more effective than MA (n = 313, MD = −1.08, 95% CI = −2.15 to −0.02, *P* = .05, *I*^2^ = 97%). In the ACE + conventional treatments versus conventional treatments analysis, there were 4 types of study designs included among 7 studies,^[35,36,39–42,45]^ and combination therapy in patients with knee OA was associated with significantly reduced pain on the VAS (n = 486, SMD = −3.00, 95% CI = −4.46 to −1.54, *P* < .0001, *I*^2^ = 97%). Due to the different study designs, we compared each pairing, and all results showed significant differences in pain intensity (Fig. 3B). In the ACE + conventional treatments versus placebo + conventional treatments analysis, 1 study^[44]^ was evaluated, the results of which also showed a significant difference between the treatment and control groups (n = 86, MD = −1.95, 95% CI = −2.03 to −1.87, *P* < .00001; Fig. 3C).

#### 3.3.2. WOMAC

**Figure 4.**
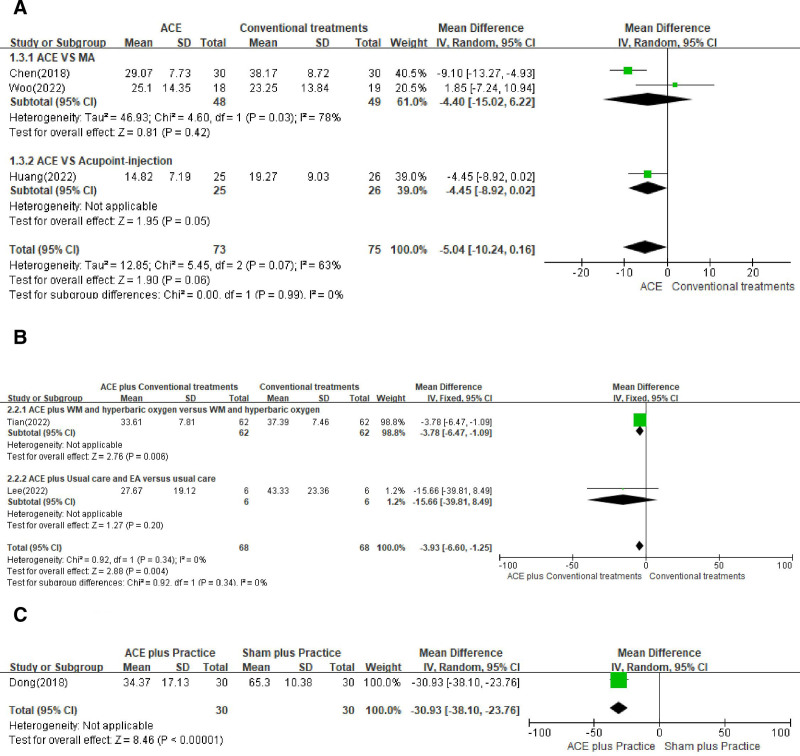
Forest plot of the Western Ontario and McMaster Universities OA Index in the comparison of ACE + conventional treatments versus conventional treatments. (A) ACE versus MA, (B) ACE + conventional treatments versus conventional treatments, and (C) ACE + practice versus sham + practice. ACE = acupoint catgut embedding, CI = confidence interval, CT = conventional treatment, EA = electroacupuncture, MA = manual acupuncture, WM = Western medicine.

A total of 148 patients from 3 studies^[17,29,48]^ were included in the ACE versus MA and ACE versus acupoint injection analysis, with no significant difference between the intervention and comparison groups (n = 148, MD = −5.04, 95% CI = −10.24 to 0.16, *P* = .06, *I*^2^ = 0%; Fig. 4A). In contrast, combination treatment^[45,55]^ was associated with a significant reduction in pain intensity based on the WOMAC results obtained at the end of the intervention period (n = 136, MD = −3.93, 95% CI = −6.60 to −1.25, *P* = .004, *I*^2^ = 0%; Fig. 4B). Only one study^[43]^ included a placebo control assessment, which showed a significant difference between the 2 groups (n = 60, MD = −30.93, 95% CI = −38.10 to −23.76, *P* < .00001; Fig. 4C).

#### 3.3.3. Total effective rate

We observed moderate differences in the reduction of symptoms in the ACE versus conventional treatments analysis^[29,32,33,48–50]^ (n = 557, RR = 1.07, 95% CI = 0.99–1.15, *P* = .07, *I*^2^ = 26%). When assessing each comparator, except MA, there was no significant difference between the 2 groups (Fig. S2, Supplemental Digital Content, https://links.lww.com/MD/Q928). When comparing combination treatments, we observed significant differences between combination treatments^[40,54,55]^ (n = 244, RR = 1.10, 95% CI = 1.02–1.20, *P* = .02, *I*^2^ = 0%; Fig. S3, Supplemental Digital Content, https://links.lww.com/MD/Q928). When we compared the effect size based on the type of comparator, there were borderline differences between the 2 groups. When we compared ACE + practice versus sham + practice,^[43]^ the pooled results were statistically significant (n = 60, RR = 1.42, 95% CI = 1.12–1.80, *P* = .004; Fig. S4, Supplemental Digital Content, https://links.lww.com/MD/Q928).

#### 3.3.4. Lysholm score

Two studies^[35,52]^ evaluated the Lysholm score in combination treatments, the results of which showed that the combination treatments were better than conventional treatments alone (ACE + warm acupuncture and ACE + arthroscopic debridement of the articular cavity, practice, and HM; n = 170, MD = 16.67, 95% CI = 11.05–22.30, *P* < .00001, *I*^2^ = 75%; Fig. S5, Supplemental Digital Content, https://links.lww.com/MD/Q928).

#### 3.3.5. Adverse events

Among the studies, 4^[33,40,45,47]^ reported no adverse events, and 18 did not record whether adverse events occurred, while only 4 RCTs^[17,29,32,44]^ reported adverse events (Table 1). The most common adverse reactions were rashes and gastrointestinal dysfunction. Pain and dizziness were considered as adverse events. No serious adverse events, however, were reported in the included studies.

#### 3.3.6. Quality of the evidence

The systematic review examined 4 different outcomes in the intervention and control groups (Tables S1–S3, Supplemental Digital Content, https://links.lww.com/MD/Q928), including VAS, WOMAC, total effective rate, and Lysholm score. All outcomes were graded as moderate, low, or very low.

## 4. Discussion

### 4.1. Principal findings

The present study is the first to investigate the correlation between treatment and effect size when ACE was performed in a clinical setting. We identified 28 studies on ACE for the treatment of knee OA encompassing 2120 participants. We examined various types of treatments used in combination with ACE, used either alone or as an adjunctive intervention, versus controls for the treatment of knee OA, as measured using the VAS, WOMAC, total effective rate, and Lysholm score. In comparison to conventional treatments alone, the meta-analysis showed significant effects of combined therapy involving ACE with conventional treatment on the primary and secondary outcomes. With regard to the sham ACE comparison, the meta-analysis of 2 trials showed that ACE significantly outperformed various forms of sham interventions, suggesting that ACE is more effective than a placebo (Figs. 3C and 4C). Regarding the comparison of ACE alone, the present meta-analysis of 5 trials showed moderate significance, suggesting a potential role for ACE to be more routinely performed as an adjunctive treatment in patients with knee OA. The risk of bias in the included RCTs was generally unclear because of the varying characteristics of ACE, resulting in a limitation in drawing reliable conclusions on the effectiveness of ACE in treating knee OA. No serious adverse events, however, were reported in the included studies.

To conduct a more comprehensive systematic review in the future, we also searched for ongoing RCTs based on the defined inclusion criteria for possible inclusion in our systematic review. We identified 2 ongoing RCTs that are eligible for future review. These studies were performed in Iran^[51]^ and Korea,^[30]^ which could reduce publication bias. Overall, the results of the present systematic review and meta-analysis suggest that ACE, when combined with other therapies, may be effective for treating knee OA.

### 4.2. Implication for research, practice, and future studies

First, in terms of implications for research, the standout requirement is for more high-quality RCTs to evaluate the effectiveness of ACE in treating knee OA. Such studies must be designed with a low risk of bias and an appropriately large sample size to be sufficiently powered. If possible, the follow-up duration should be longer, ideally 6 to 12 months, after the end of treatment. In ACE, thread or catgut is inserted into the body for long-term stimulation of the acupoint, which is different from MA.^[13]^ In the present study, we tried to assess the treatment frequency and follow-up treatment effects; however, only 4 studies were assessed,^[45,47,52,55]^ which used different outcomes, and we could not compare the outcomes. We recommend that this be evaluated in future studies. Additionally, an economic evaluation of ACE for the treatment of knee OA needs to be conducted, as 1 study evaluated the economic impact of ACE for obesity, which indicated that ACE was less costly in obesity management.^[58]^ In future clinical studies, therefore, it would be valuable to conduct cost-effectiveness and non-inferiority trials. Second, the results of the present meta-analysis indicated that the most common acupoint was GB34 because GB34 is an acupoint of the gallbladder meridian. Based on this mechanism, with ACE inserted into the gall bladder meridian acupoint, the integrin-linked kinase and catenin signal pathway can be regulated.^[59]^ However, most studies have assessed outcomes using questionnaires, without specific blood test results, as only adverse effects were assessed. Future clinical studies should assess blood tests for specific outcomes. Finally, various countries have used ACE, such as Iran, Korea, and China, although there are some differences between these countries. For example, in China, the treatment uses the acupoint or sinew meridian,^[29]^ whereas, in Korea, it uses the medial collateral ligament, pes anserinus, lateral joint line, or acupoint.^[17,45]^ Furthermore, each country uses different materials, such as polydioxanone, polyglactin acid, and catgut. We could not perform a meta-analysis based on the characteristics of the materials because of the small number of studies included. In future studies, therefore, international clinical trials should be performed, and the effectiveness of these different methods should be compared or examined based on the thread material.

### 4.3. Strengths and limitations

The present systematic review and meta-analysis had several strengths: we searched either international or Chinese and Korean databases to identify the included articles; we searched for ongoing trials to determine the status of ACE and used common clinical evaluation tools to assess knee OA; and we provided a comprehensive review of the effectiveness of ACE to consider possible sources of heterogeneity. The quality of evidence for each outcome was also evaluated using GradePro.

The present study did have several limitations. First, low-quality RCTs were included, and more than half of the trials did not blind the practitioners due to the nature of the ACE intervention. These low-quality studies could have led to inaccurate results. Second, the meta-analysis had heterogeneity among the included studies, although we used a different study design to reduce heterogeneity. The sample size, however, was small based on the study design, the heterogeneity of which could not be resolved. Third, although we included databases from various countries, only 2 studies were performed in Korea. These factors may have led to publication bias. This bias may result in the present study being considered less informative, with results that may be difficult to generalize.

## 5. Conclusions

Finally, we comprehensively evaluated the effects of ACE in combination with and separate from other treatments. We found that ACE had a moderate effect compared to other therapies for treating knee OA. ACE combined with other therapies is better effect to treat Knee OA than ACE alone. Although the present systemic review and meta-analysis provided low-quality evidence and heterogeneity, it can be a helpful guide for public health practices and future clinical studies.

## Author contributions

**Conceptualization:** HuiYan Zhao, Chang-Hyun Han.

**Formal analysis:** HuiYan Zhao, Yan Liu, Changsop Yang.

**Methodology:** HuiYan Zhao, Yan Liu, Changsop Yang.

**Supervision:** HuiYan Zhao, Chang-Hyun Han.

**Writing – original draft:** HuiYan Zhao.

**Writing – review & editing:** HuiYan Zhao, JaeWan Lee, Chang-Hyun Han.

## Supplementary Material


