# Supplementary material for: Efficacy and safety of acupoint catgut embedding for knee osteoarthritis: A systematic review and meta-analysis
Source: Medicine (Baltimore). 2025 Dec 19;104(51):e46676. doi: 10.1097/MD.0000000000046676 (PMC12727354; doi:10.1097/MD.0000000000046676)
Supplement: Supplementary file 1 [file medi-104-e46676-s001.pdf]

Supplementary Figure 1 The detail of risk of bias for each study

| | Random sequence generation (selection bias) | Allocation concealment (selection bias) | Blinding of participants and personnel (performance bias) | Blinding of outcome assessment (detection bias) | Incomplete outcome data (attrition bias) | Selective reporting (reporting bias) | Other bias |
|---|---|---|---|---|---|---|---|
| Bahrami(2023) | + | + | − | ? | ? | + | + |
| Chen(2018) | + | + | − | ? | + | + | ? |
| Cheng(2018) | + | + | − | ? | + | + | ? |
| Dong(2018) | + | + | − | ? | + | + | ? |
| Guo(2016) | + | ? | − | ? | + | + | ? |
| He(2021) | ? | ? | − | ? | + | + | ? |
| Hou(2010) | ? | ? | − | ? | + | + | ? |
| Huang(2021) | ? | ? | − | ? | + | + | ? |
| Huang(2022) | ? | ? | − | ? | + | + | ? |
| Jang(2023) | + | + | − | + | ? | + | + |
| Lee(2022) | + | + | − | + | + | + | + |
| Lei(2022) | ? | ? | − | ? | + | + | ? |
| Lin(2020) | + | + | − | ? | + | + | ? |
| Mai(2014) | + | − | − | ? | + | + | ? |
| Mo(2017) | + | ? | − | ? | + | + | ? |
| Pan(2020) | + | + | − | ? | + | + | ? |
| Shu(2022) | ? | ? | − | ? | + | + | ? |
| Tian(2022) | + | + | − | ? | + | + | ? |
| Wang(2021) 1 | ? | ? | − | ? | + | + | ? |
| Wang(2021) 2 | + | + | − | − | + | + | + |
| Wang(2021) 3 | ? | ? | − | ? | + | + | ? |
| Wang(2021) 4 | ? | ? | − | ? | + | + | ? |
| Woo(2022) | + | + | − | + | + | + | + |
| Yang(2016) | + | ? | − | ? | + | + | ? |
| Yang(2017) | + | ? | − | ? | + | + | ? |
| Yang(2023) | ? | ? | − | ? | + | + | ? |
| Yu(2022) | ? | ? | − | ? | + | + | ? |
| Zhang(2022) | + | + | − | ? | + | + | ? |

Supplementary Figure 2: Forest plot of the total effective rate in the comparison ACE versus Conventional treatments

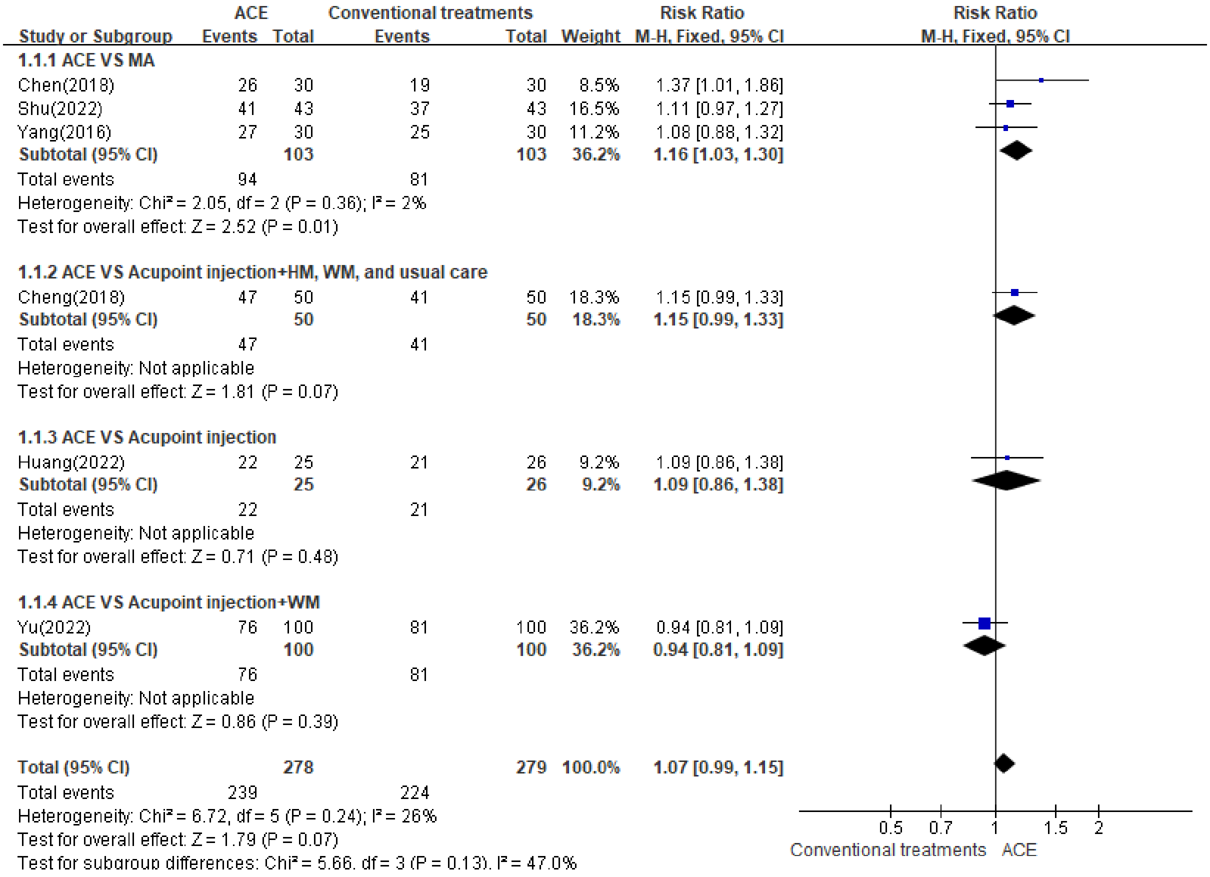


Abbreviation: ACE: Acupoint Catgut Embedding; CT: conventional treatments; HM: Herbal Medicine; MA: Manual acupuncture; WM: Western Medicine

Supplementary Figure 3: Forest plot of the total effective rate in the comparison ACE plus Conventional treatments versus Conventional treatments

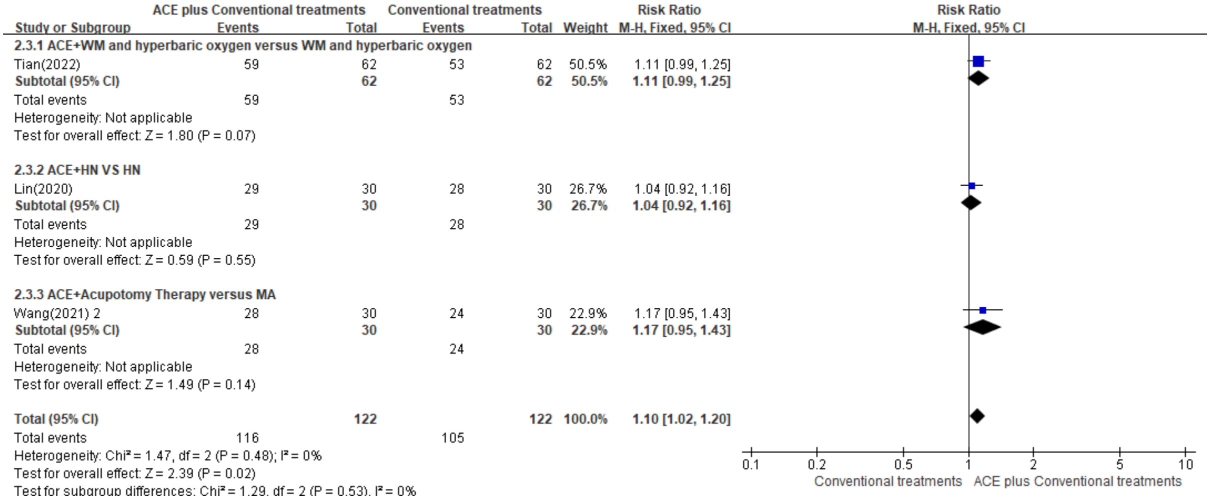


Abbreviation: ACE: Acupoint Catgut Embedding; CT: conventional treatments; HN: Heated needle; MA: Manual acupuncture; WM: Western Medicine

Supplementary Figure 4: Forest plot of the total effective rate in the comparison ACE plus practice versus sham plus practice.

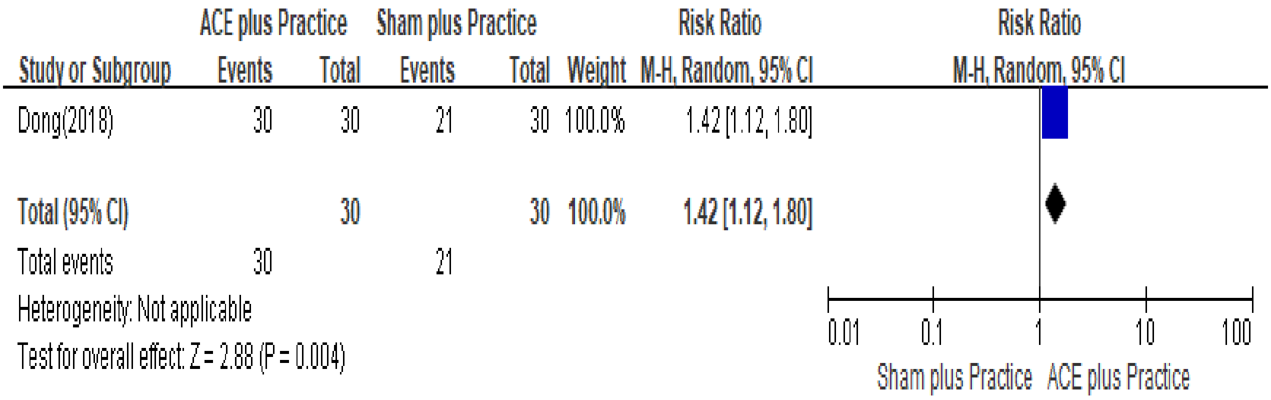


Abbreviation: ACE: Acupoint Catgut Embedding

Supplementary Figure 5: Forest plot of the Lysholm Score in the comparison ACE plus Conventional treatments versus Conventional treatments

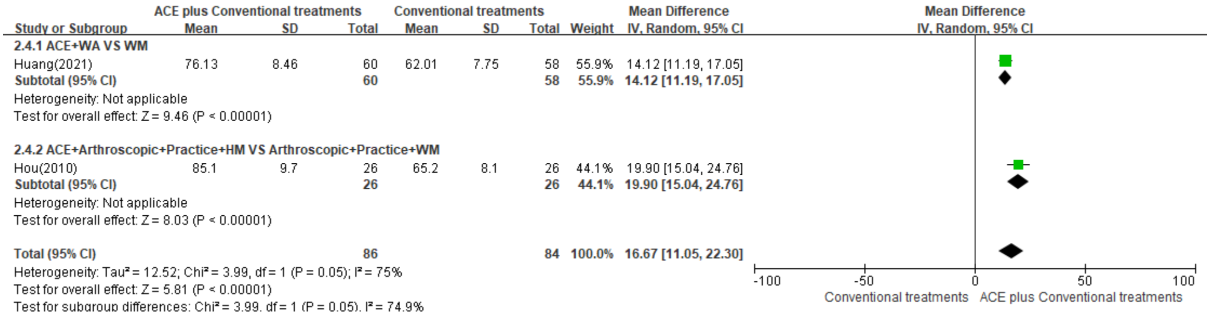


Abbreviation: ACE: Acupoint Catgut Embedding; CT: conventional treatments; HM: Herbal medicine; WA: Warm acupuncture; WM: Western Medicine

Supplementary Table 1. Quality of evidence assessment for ACE versus conventional treatments

| Certainty assessment | | | | | | | NO. of participants | | Effect | | Certainty | importance |
|---|---|---|---|---|---|---|---|---|---|---|---|---|
| No of studies | Study design | Risk of bias | Inconsistency | Indirectness | imprecision | Other consideration | ACE | Conventional treatments | Relative (95%CI) | Absolute (95%CI) | | |
| VAS: ACE versu MA | | | | | | | | | | | | |
| 5 | randomised trials | serious[a] | very serious[e] | not serious | not serious | none | 156 | 157 | - | MD **1.08 lower** (2.15 lower to 0.02 lower) | ⊕◯◯◯ Very low | CRITICAL |
| WOMAC: ACE versus Conventional treatments | | | | | | | | | | | | |
| 3 | randomised trials | serious[a] | serious[d] | not serious | not serious | none | 73 | 75 | - | MD **5.04 lower** (10.24 lower to 0.16 higher) | ⊕⊕◯◯ Low | CRITICAL |
| WOMAC: ACE versus MA | | | | | | | | | | | | |
| 2 | randomised trials | serious[a] | serious[e] | serious[b] | not serious | none | 48 | 49 | - | MD **4.4 lower** (15.02 lower to 6.22 higher) | ⊕◯◯◯ Very low | CRITICAL |
| WOMAC: ACE versus Acupoint-injection | | | | | | | | | | | | |
| 1 | randomised trials | serious[a] | not serious | serious[b] | serious[b] | none | 25 | 26 | - | MD **4.45 lower** (8.92 lower to 0.02 higher) | ⊕◯◯◯ Very low | CRITICAL |
| Total effective rate: ACE versus Conventional treatments | | | | | | | | | | | | |
| 6 | randomised trials | serious[a] | not serious | not serious | serious[C] | none | 239/278 (86.0%) | 224/279 (80.3%) | **RR 1.07 (0.99 to 1.15)** | **56 more per 1,000 (from 8 fewer to 120 more)** | ⊕⊕⊕◯ Moderate | IMPORTANT |
| Total effective rate: ACE versus MA | | | | | | | | | | | | |
| 3 | randomised trials | serious[a] | not serious | not serious | not serious | none | 94/103 (91.3%) | 81/103 (78.6%) | **RR 1.16** (1.03 to 1.30) | **126 more per 1,000** (from 24 more to 236 more) | ⊕⊕⊕◯ Moderate | IMPORTANT |
| Total effective rate: ACE versus Acupoint injection plus HM, WM, and usual care | | | | | | | | | | | | |
| 1 | randomised trials | serious[a] | not serious | not serious | serious[b,c] | none | 47/50 (94.0%) | 41/50 (82.0%) | **RR 1.15** (0.99 to 1.33) | **123 more per 1,000** (from 8 | ⊕⊕◯◯ Low | IMPORTANT |

| | | | | | | | | | | | | |
|---|---|---|---|---|---|---|---|---|---|---|---|---|
| | | | | | | | | | | fewer to 271 more) | | |
| Total effective rate: ACE versus Acupoint injection | | | | | | | | | | | | |
| 1 | randomised trials | serious[a] | not serious | not serious | serious[b,c] | none | 22/25 (88.0%) | 21/26 (80.8%) | **RR 1.09** (0.86 to 1.38) | **73 more per 1,000** (from 113 fewer to 307 more) | ⊕⊕◯◯ Low | IMPORTANT |
| Total effective rate: ACE versus Acupoint injection plus WM | | | | | | | | | | | | |
| **1** | randomised trials | serious[a] | serious[d] | not serious | serious[c] | none | 76/100 (76.0%) | 81/100 (81.0%) | **RR 0.94 (0.81 to 1.09)** | **49 fewer per 1,000 (from 154 fewer to 73 more)** | ⊕◯◯◯ Very low | IMPORTANT |

a. "Blinding of outcome assessment" is high; b. Sample size was lower than 100; c. Risk ratio was included 0; d. I2 is higher than 50 and lower than 75; e. I2 is higher than 75 CI: confidence interval; MD: mean difference; RR: risk ratio

Supplementary Table 2. Quality of evidence assessment for ACE plus Conventional treatments versus Conventional treatments

| Certainty assessment | | | | | | | NO. of participants | | Effect | | Certainty | importance |
|---|---|---|---|---|---|---|---|---|---|---|---|---|
| No of studies | Study design | Risk of bias | Inconsistency | Indirectness | imprecision | Other consideration | ACE | Conventional treatments | Relative (95%CI) | Absolute (95%CI) | | |
| VAS: ACE plus Conventional treatments versus Conventional treatments | | | | | | | | | | | | |
| 7 | randomised trials | serious[a] | very serious[b] | not serious | not serious | none | 244 | 242 | - | SMD **3 lower** (4.46 lower to 1.54 lower) | ⊕◯◯◯ Very low | CRITICAL |
| VAS: ACE plus WM versus WM | | | | | | | | | | | | |
| 1 | randomised trials | serious[a] | not serious | not serious | serious[c] | none | 31 | 31 | - | SMD **5.42 lower** (6.52 lower to 4.32 lower) | ⊕⊕◯◯ Low | CRITICAL |
| VAS: ACE plus WA versus WM | | | | | | | | | | | | |
| 2 | randomised trials | serious[a] | not serious | not serious | not serious | none | 111 | 109 | - | SMD **4.14 lower** (4.71 lower to 3.57 lower) | ⊕⊕⊕◯ Moderate | CRITICAL |
| VAS: ACE plus Acupotomy versus MA | | | | | | | | | | | | |
| 3 | randomised trials | serious[a] | serious[b] | not serious | not serious | none | 96 | 96 | - | SMD **1.95 lower** (3.71 | ⊕⊕◯◯ Low | CRITICAL |

| | | | | | | | | | | | | |
|---|---|---|---|---|---|---|---|---|---|---|---|---|
| | | | | | | | | | | lower to 0.19 lower) | | |
| VAS: ACE plus Usual care and EA versus usual care | | | | | | | | | | | | |
| 1 | randomised trials | serious[a] | not serious | not serious | serious[c] | none | 6 | 6 | - | SMD **1.39 lower** (2.71 lower to 0.07 lower) | ⊕⊕◯◯ Low | CRITICAL |
| WOMAC: ACE plus Conventional treatments versus Conventional treatments | | | | | | | | | | | | |
| 2 | randomised trials | serious[a] | not serious | not serious | not serious | none | 68 | 68 | - | MD **3.93 lower** (6.6 lower to 1.25 lower) | ⊕⊕⊕◯ Moderate | CRITICAL |
| WOMAC: ACE plus WM and hyperbaric oxygen versus WM and hyperbaric oxygen | | | | | | | | | | | | |
| 1 | randomised trials | serious[a] | not serious | not serious | not serious | none | 62 | 62 | - | MD **3.78 lower** (6.47 lower to 1.09 lower) | ⊕⊕⊕◯ Moderate | CRITICAL |
| WOMAC: ACE plus Usual care and electro-acupuncture versus usual care | | | | | | | | | | | | |
| 1 | randomised trials | serious[a] | not serious | not serious | serious[c] | none | 6 | 6 | - | MD **15.66 lower** (39.81 lower to 8.49 higher) | ⊕⊕◯◯ Low | CRITICAL |
| Total effective rate: ACE plus Conventional treatments versus Conventional treatments | | | | | | | | | | | | |
| 3 | randomised trials | serious[a] | not serious | not serious | not serious | none | 116/122 (95.1%) | 105/122 (86.1%) | **RR 1.10** (1.02 to 1.20) | **86 more per 1,000** (from 17 more to 172 more) | ⊕⊕⊕◯ Moderate | IMPORTANT |
| Total effective rate: ACE plus WM and hyperbaric oxygen versus WM and hyperbaric oxygen | | | | | | | | | | | | |
| 1 | randomised trials | serious[a] | not serious | not serious | serious[d] | none | 59/62 (95.2%) | 53/62 (85.5%) | **RR 1.11** (0.99 to 1.25) | **94 more per 1,000** (from 9 fewer to 214 more) | ⊕⊕◯◯ Low | IMPORTANT |
| Total effective rate: ACE plus HN VS HN | | | | | | | | | | | | |
| 1 | randomised trials | serious[a] | not serious | not serious | serious[c,d] | none | 29/30 (96.7%) | 28/30 (93.3%) | **RR 1.04** (0.92 to 1.16) | **37 more per 1,000** (from 75 fewer to 149 more) | ⊕⊕◯◯ Low | IMPORTANT |
| Total effective rate: ACE plus Acupotomy Therapy versus MA | | | | | | | | | | | | |

| | | | | | | | | | | | | |
|---|---|---|---|---|---|---|---|---|---|---|---|---|
| 1 | randomised trials | serious[a] | not serious | not serious | serious[c,d] | none | 28/30 (93.3%) | 24/30 (80.0%) | **RR 1.17** (0.95 to 1.43) | **136 more per 1,000** (from 40 fewer to 344 more) | ⊕⊕◯◯ Low | IMPORTANT |
| Lysholm Score: ACE plus Conventional treatments versus Conventional treatments | | | | | | | | | | | | |
| 2 | randomised trials | serious[a] | not serious | not serious | not serious | none | 86 | 84 | - | MD **16.67 higher** (11.05 higher to 22.3 higher) | ⊕⊕⊕◯ Moderate | IMPORTANT |
| Lysholm Score: ACE plus WA VS WM | | | | | | | | | | | | |
| 1 | randomised trials | serious[a] | not serious | not serious | not serious | none | 60 | 58 | - | MD **14.12 higher** (11.19 higher to 17.05 higher) | ⊕⊕⊕◯ Moderate | IMPORTANT |
| Lysholm Score: ACE plus Arthroscopic, Practice, and HM VS Arthroscopic, Practice, and HM | | | | | | | | | | | | |
| 1 | randomised trials | serious[a] | not serious | not serious | serious[c] | none | 26 | 26 | - | MD **19.9 higher** (15.04 higher to 24.76 higher) | ⊕⊕◯◯ Low | IMPORTANT |

ACE: Acupoint Catgut Embedding; a. Blinding of outcome assessment is high; b. I2 is higher than 75; c. Sample size is lower than 100; d. Risk ratio was included 0; EA: Electro-acupuncture; HM: Herbal Medicine; HN: Heated needle; MA: Manual acupuncture; VAS: Visual Analog Scale; WA: Warm acupuncture; WOMAC: Western Ontario and McMaster Universities OA Index; WM: Western Medicine

## Supplementary Table 3. Quality of evidence assessment for ACE plus Conventional treatments versus Sham plus Conventional treatments

| Certainty assessment | | | | | | | NO. of participants | | Effect | | Certainty | importance |
|---|---|---|---|---|---|---|---|---|---|---|---|---|
| No of studies | Study design | Risk of bias | Inconsistency | Indirectness | imprecision | Other consideration | ACE plus Conventional treatments | Sham ACE plus Conventional treatments | Relative (95%CI) | Absolute (95%CI) | | |
| VAS: ACE plus Practice versus sham plus Practice | | | | | | | | | | | | |
| 1 | randomised trials | serious[a] | not serious | not serious | serious[b] | none | 43 | 43 | - | MD 1.95 lower (2.03 lower to 1.87 lower) | ⊕⊕◯◯ Low | CRITICAL |
| WOMAC: ACE plus WM versus sham plus WM | | | | | | | | | | | | |
| 1 | randomised trials | serious[a] | not serious | not serious | serious[b] | none | 30 | 30 | - | MD 30.93 lower (38.1 | ⊕⊕◯◯ Low | CRITICAL |

| | | | | | | | | | | | | |
|---|---|---|---|---|---|---|---|---|---|---|---|---|
| | | | | | | | | | | lower to 23.76 lower) | | |
| Total effective rate: ACE plus Practice versus sham plus Practice | | | | | | | | | | | | |
| 1 | randomised trials | serious[a] | not serious | not serious | serious[b] | none | 30/30 (100.0%) | 21/30 (70.0%) | RR 1.42 (1.12 to 1.80) | 294 more per 1,000 (from 84 more to 560 more) | ⊕⊕◯◯ Low | IMPORTANT |

ACE: acupoint catgut embedding; a. Blinding of outcome assessment is high; b. sample size is smaller than 100; CI: confidence interval; MD: mean difference; RR: risk ratio; VAS: Visual Analog Scale; WOMAC: Western Ontario and McMaster Universities OA Index; WM: Western Medicine
